# A new species of the genus *Stylicletodes* Lang, 1936 (Copepoda, Harpacticoida, Cletodidae) from South Korea

**DOI:** 10.3897/BDJ.10.e90590

**Published:** 2022-10-19

**Authors:** Kyuhee Cho, Jong Guk Kim, Jimin Lee

**Affiliations:** 1 Marine Ecosystem Research Center, Korea Institute of Ocean Science & Technology, Busan, Republic of Korea Marine Ecosystem Research Center, Korea Institute of Ocean Science & Technology Busan Republic of Korea; 2 Division of Zoology, Honam National Institute of Biological Resources, Mokpo, Republic of Korea Division of Zoology, Honam National Institute of Biological Resources Mokpo Republic of Korea

**Keywords:** Crustacea, meiofauna, *Stylicletodestrifidus* sp. nov., Yellow Sea, South Sea

## Abstract

**Background:**

Marine benthic harpacticoid copepods are poorly known in Korea due to the difficulty in obtaining specimens. Currently, the genus *Stylicletodes* Lang, 1936, which is known to occur in subtidal sediments, has not been reported in this area so far. During surveys on the subtidal meiofauna, we found a new species of *Stylicletodes* from several subtidal muddy sediments in the Yellow Sea and South Sea.

**New information:**

In this study, we describe both sexes of a new species of *Stylicletodes* collected from the Yellow Sea and South Sea of Korea. *Stylicletodestrifidus* sp. nov. differs from its congeners in the following characteristics: the trifid rostrum, relative length ratio of the endopods to exopods on legs 1–4, reduced armature formulae on legs 3–4, constricted shape at mid-length of the anal somite, and structure of the sexually dimorphic male leg 3 with a two-segmented endopod. The new species underwent loss of the maxilliped, which is very rare in harpacticoids and is probably an important clue for the phylogeny of the species of *Stylicletodes*.

## Introduction

Benthic harpacticoids are very diverse and are widespread worldwide (WoRMS Editorial Board 2022). Many researchers have devoted considerable effort to the study of the diversity of benthic harpacticoids in Korean waters, which have the greatest number of species per unit area (Costello et al. 2010); currently, ~ 200 marine harpacticoids species have been discovered in various habitats in this region (Chang 2010, Lee et al. 2012, Song et al. 2012, Choi 2022). Nevertheless, many Korean harpacticoid species remain unidentified (Back and Lee 2014, Kim 2014, Kim 2017) and subtidal harpacticoid copepods are particularly poorly known due to the difficulty in obtaining specimens. Recent efforts have begun to reveal the diversity of subtidal marine benthic copepods (> 50 m depth) around the Korean Peninsula (Huys and Lee 2018, Lee and Huys 2019, Kim and Lee 2019, Kim et al. 2021b).

The family Cletodidae T. Scott, 1905 is a benthic harpacticoid taxon with about 150 species in 38 genera (George 2020, George 2021, Walter and Boxshall 2022), including species of the *Ceratonotus*-group *sensu* Conroy-Dalton, 2001 formerly placed in Ancorabolinae Sars, 1909 (George 2020). Seventeen species distributed in nine genera of Cletodidae have been reported from Korea: *Enhydrosoma* Boeck, 1872 (seven species); *Limnocletodes* Borutzky, 1926 and *Paracrenhydrosoma* Gee, 1999 (two species each); and *Cletodes* Brady, 1872, *Dendropsyllus* Conroy-Dalton, 2003, *Dimorphipodia* Lee & Huys, 2019, *Geehydrosoma* Kim, Trebukhova, Lee & Karanovic, 2014, *Kollerua* Gee, 1994, and *Strongylacron* Gee & Huys, 1996 (one species each) (Lee and Chang 2007, Kim 2013, Kim 2014, Kim et al. 2014, Song et al. 2014, Karanovic et al. 2015, Kim et al. 2016, Lee and Huys 2019). However, the identity of five cletodid harpacticoids recorded by Kim (2013) and Kim (2014) remains controversial (Huys and Lee 2018, Lee and Huys 2019, Song et al. 2020, Kim et al. 2021a): *Paracrenhydrosomakarlingi* (Lang, 1965) (as *Acrenhydrosomakarlingi* Lang, 1965 in Kim (2014)), *Cletodesdentatus* Wells & Lao, 1987, *Enhydrosomacurticauda* Boeck, 1872, *E.latipes* (A. Scott, 1909) and *E.longicauda* Marinov & Apostolov, 1983. On the other hand, the existence of species of the genus *Stylicletodes* Lang, 1936 was recognized in the Korean seas by Kim et al. (2014) and Karanovic et al. (2015) who used it as a comparison group in their genetic analyses of species of *Enhydrosoma*. However, the taxonomic description of the species of *Stylicletodes* has not been published so far and this genus remains unreported in Korea.

A new species of the genus *Stylicletodes* was found in sublittoral samples taken from the Yellow Sea and South Sea during surveys on the diversity of benthic harpacticoids from Korea. Here we give a detailed description of the new species.

## Materials and methods

**Figure 1.**
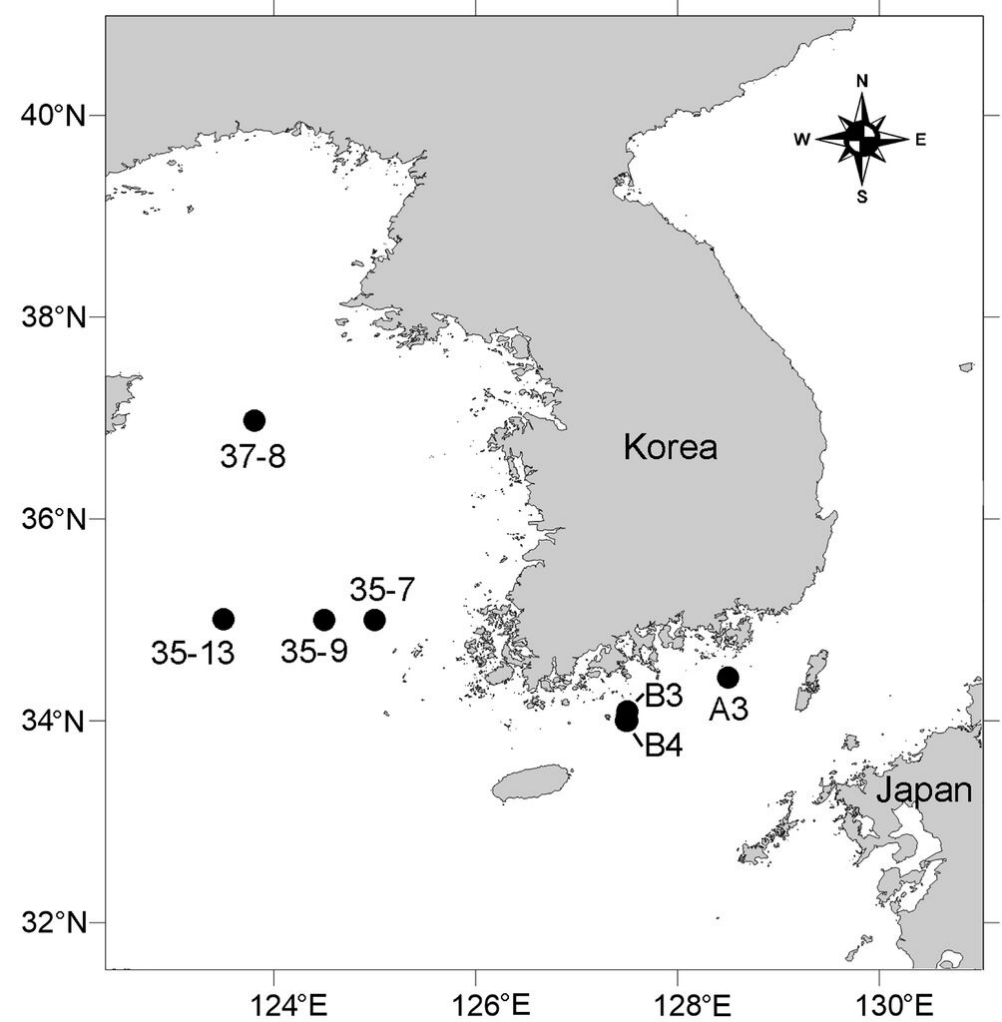
Position of the sampling stations in the Yellow Sea and South Sea of Korea.

Samplings were conducted at several stations in the Yellow Sea and South Sea (Fig. 1; Table 1) on board R/V Eardo (Korea Institute of Ocean Science & Technology (KIOST)). Sediments were taken with a Smith-McIntyre grab (0.1 m^2^) and the upper surface (> 5 cm) of the sediments was subsampled using a trowel for qualitative analyses. A solution of 7.5% magnesium chloride (MgCl_2_) was added to the subsample and fixed after 30 minutes with a 10% formalin/seawater solution. Meiofauna was separated from the coarser grains of sediment through the centrifugation method with LUDOX® HS-40 (Burgess 2001). Copepods were sorted under a stereomicroscope (M165 C; Leica, Germany) and preserved in 5% formalin. Specimens were dissected with tungsten needles, mounted in lactophenol:glycerine (1:3) or Fluoromount-G (SouthernBiotech, USA) on glass slides or H-S slides (Double slide plate, BSDS-011R; Biosolution, Republic of Korea) (cf. Shirayama et al. 1993) and sealed with transparent nail varnish. A differential interference contrast (DIC) light microscope (DM2500; Leica, Germany) with a drawing tube was used to make morphometric measurements and illustrations. The mouth appendages were photographed with a CCD camera (DP26; Olympus, Japan) mounted on a differential interference contrast microscope (BX53; Olympus, Japan).

The total body length of individuals was measured dorsally from the anterior tip of the rostrum to the posterior end of the caudal rami in the dorsal view and was sometimes calculated as the sum of the mid-dorsal lengths of each somite measured in the lateral view, not considering the various degrees of telescoping of somites. The length/width ratio (L/W) of the anal somite was calculated by measuring the length along the middle in ventral view and the largest width at the anterior part. The L/W of the caudal rami was calculated by measuring the length along the outer margin in the dorsal view and the widest part at the insertion point of lateral setae I and II. The L/W of the P5 exopod was calculated by measuring the length along the outer margin and width above the insertion of the proximal outer seta. Scale bars in the figures are indicated in micrometers (μm).

Type material was deposited in the Marine Biodiversity Institute of Korea (MABIK), Seocheon, Republic of Korea and additional materials were stored at the Marine Interstitial fauna Resources Bank (MInRB) in KIOST, Busan, Republic of Korea.

The morphological terminology used in the text and figure legends follows Huys et al. (1996). Abbreviations are ae = aesthetasc; apo = apophysis; P1–P6 = first-to-sixth thoracic leg; exp(enp)-1(2, 3) = to denote the proximal (middle, distal) segment of a three-segmented ramus.

## Taxon treatments

### Stylicletodes trifidus

Cho, Kim & Lee sp. n.

B1A73003-DF6B-5769-B160-346A49D935CC

6E09685D-89F1-4782-BC39-75E8ABDE2EA9

Stylicletodes Lang, 1936
Type species: *Stylicletodeslongicaudatus* (Brady, 1880)

#### Materials

- **Type status:** Holotype. **Occurrence:** individualCount: 1; sex: female; lifeStage: adult; preparations: preserved in 95% ethanol; occurrenceID: 7790343C-DDD5-54FE-897F-B6B002B5DBAD; **Taxon:** kingdom: Animalia; phylum: Arthropoda; class: Copepoda; order: Harpacticoida; family: Cletodidae; genus: Stylicletodes; specificEpithet: trifidus; taxonRank: species; scientificNameAuthorship: Cho, Kim & Lee; **Location:** higherGeography: East Asia; waterBody: Yellow sea; country: Korea; countryCode: KR; verbatimDepth: 77.2 m; locationRemarks: stn 37-8; verbatimLatitude: 36°58'24.66"N; verbatimLongitude: 123°48'39.72"E; decimalLatitude: 36.97351667; decimalLongitude: 123.81103333; **Identification:** identifiedBy: Cho, Kim & Lee; dateIdentified: 2022; **Event:** samplingProtocol: Smith-McIntyre grab; eventDate: 24/04/2019; **Record Level:** institutionID: MABIK CR00252667; institutionCode: Marine Biodiversity Institute of Korea (MABIK); basisOfRecord: PreservedSpecimen
- **Type status:** Paratype. **Occurrence:** individualCount: 1; sex: male; lifeStage: adult; preparations: dissected on 4 slides; occurrenceID: 8984821E-EBCE-51F6-9C49-DB90C0947C58; **Taxon:** kingdom: Animalia; phylum: Arthropoda; class: Copepoda; order: Harpacticoida; family: Cletodidae; genus: Stylicletodes; specificEpithet: trifidus; taxonRank: species; scientificNameAuthorship: Cho, Kim & Lee; **Location:** higherGeography: East Asia; waterBody: Yellow sea; country: Korea; countryCode: KR; verbatimDepth: 77.2 m; locationRemarks: stn 37-8; verbatimLatitude: 36°58'24.66"N; verbatimLongitude: 123°48'39.72"E; decimalLatitude: 36.97351667; decimalLongitude: 123.81103333; **Identification:** identifiedBy: Cho, Kim & Lee; dateIdentified: 2022; **Event:** samplingProtocol: Smith-McIntyre grab; eventDate: 24/04/2019; **Record Level:** institutionID: MABIK CR00252670; institutionCode: Marine Biodiversity Institute of Korea (MABIK); basisOfRecord: PreservedSpecimen
- **Type status:** Paratype. **Occurrence:** individualCount: 1; sex: female; lifeStage: adult; preparations: dissected on 11 slides; occurrenceID: 2CD9F77A-1962-5EAA-BA24-4EA8D8C9955D; **Taxon:** kingdom: Animalia; phylum: Arthropoda; class: Copepoda; order: Harpacticoida; family: Cletodidae; genus: Stylicletodes; specificEpithet: trifidus; taxonRank: species; scientificNameAuthorship: Cho, Kim & Lee; **Location:** higherGeography: East Asia; waterBody: Yellow sea; country: Korea; countryCode: KR; verbatimDepth: 77.2 m; locationRemarks: stn 37-8; verbatimLatitude: 36°58'24.66"N; verbatimLongitude: 123°48'39.72"E; decimalLatitude: 36.97351667; decimalLongitude: 123.81103333; **Identification:** identifiedBy: Cho, Kim & Lee; dateIdentified: 2022; **Event:** samplingProtocol: Smith-McIntyre grab; eventDate: 24/04/2019; **Record Level:** institutionID: MABIK CR00252669; institutionCode: Marine Biodiversity Institute of Korea (MABIK); basisOfRecord: PreservedSpecimen
- **Type status:** Paratype. **Occurrence:** individualCount: 1; sex: male; lifeStage: adult; preparations: preserved in 95% ethanol; occurrenceID: 60FF350A-DDD9-551D-ADB0-06FF2AD80EF2; **Taxon:** kingdom: Animalia; phylum: Arthropoda; class: Copepoda; order: Harpacticoida; family: Cletodidae; genus: Stylicletodes; specificEpithet: trifidus; taxonRank: species; scientificNameAuthorship: Cho, Kim & Lee; **Location:** higherGeography: East Asia; waterBody: Yellow sea; country: Korea; countryCode: KR; verbatimDepth: 77.2 m; locationRemarks: stn 37-8; verbatimLatitude: 36°58'24.66"N; verbatimLongitude: 123°48'39.72"E; decimalLatitude: 36.97351667; decimalLongitude: 123.81103333; **Identification:** identifiedBy: Cho, Kim & Lee; dateIdentified: 2022; **Event:** samplingProtocol: Smith-McIntyre grab; eventDate: 24/04/2019; **Record Level:** institutionID: MABIK CR00252668; institutionCode: Marine Biodiversity Institute of Korea (MABIK); basisOfRecord: PreservedSpecimen
- **Type status:** Paratype. **Occurrence:** individualCount: 1; sex: female; lifeStage: adult; preparations: dissected on 3 slides; occurrenceID: DE8EB9F0-2FD1-5FA0-9779-7A3A6F2FD586; **Taxon:** kingdom: Animalia; phylum: Arthropoda; class: Copepoda; order: Harpacticoida; family: Cletodidae; genus: Stylicletodes; specificEpithet: trifidus; taxonRank: species; scientificNameAuthorship: Cho, Kim & Lee; **Location:** higherGeography: East Asia; waterBody: Yellow sea; country: Korea; countryCode: KR; verbatimDepth: 77.2 m; locationRemarks: stn 37-8; verbatimLatitude: 36°58'24.66"N; verbatimLongitude: 123°48'39.72"E; decimalLatitude: 36.97351667; decimalLongitude: 123.81103333; **Identification:** identifiedBy: Cho, Kim & Lee; dateIdentified: 2022; **Event:** samplingProtocol: Smith-McIntyre grab; eventDate: 24/04/2019; **Record Level:** basisOfRecord: PreservedSpecimen
- **Type status:** Paratype. **Occurrence:** individualCount: 3; sex: male; lifeStage: adult; preparations: dissected each on 3 slides; occurrenceID: E3AA6628-F9BC-5586-A548-61E148410951; **Taxon:** kingdom: Animalia; phylum: Arthropoda; class: Copepoda; order: Harpacticoida; family: Cletodidae; genus: Stylicletodes; specificEpithet: trifidus; taxonRank: species; scientificNameAuthorship: Cho, Kim & Lee; **Location:** higherGeography: East Asia; waterBody: Yellow sea; country: Korea; countryCode: KR; verbatimDepth: 77.2 m; locationRemarks: stn 37-8; verbatimLatitude: 36°58'24.66"N; verbatimLongitude: 123°48'39.72"E; decimalLatitude: 36.97351667; decimalLongitude: 123.81103333; **Identification:** identifiedBy: Cho, Kim & Lee; dateIdentified: 2022; **Event:** samplingProtocol: Smith-McIntyre grab; eventDate: 24/04/2019; **Record Level:** basisOfRecord: PreservedSpecimen
- **Type status:** Other material. **Occurrence:** individualCount: 1; sex: female; lifeStage: adult; preparations: dissected on 3 slides; occurrenceID: 1ED7806C-E517-54D0-A0AE-08DC1B6AA73A; **Taxon:** kingdom: Animalia; phylum: Arthropoda; class: Copepoda; order: Harpacticoida; family: Cletodidae; genus: Stylicletodes; specificEpithet: trifidus; taxonRank: species; scientificNameAuthorship: Cho, Kim & Lee; **Location:** higherGeography: East Asia; waterBody: Yellow sea; country: Korea; countryCode: KR; verbatimDepth: 88.2 m; locationRemarks: stn 35-7 #2; verbatimLatitude: 34°59'51.00"N; verbatimLongitude: 125°00'04.2"E; decimalLatitude: 34.9975; decimalLongitude: 125.00116667; **Identification:** identifiedBy: Cho, Kim & Lee; dateIdentified: 2022; **Event:** samplingProtocol: Smith-McIntyre grab; eventDate: 29/08/2020; **Record Level:** basisOfRecord: PreservedSpecimen
- **Type status:** Other material. **Occurrence:** individualCount: 1; sex: male; lifeStage: adult; preparations: preserved in 95% ethanol; occurrenceID: FA0771F8-1D46-5620-849F-40976B6E7E87; **Taxon:** kingdom: Animalia; phylum: Arthropoda; class: Copepoda; order: Harpacticoida; family: Cletodidae; genus: Stylicletodes; specificEpithet: trifidus; taxonRank: species; scientificNameAuthorship: Cho, Kim & Lee; **Location:** higherGeography: East Asia; waterBody: Yellow sea; country: Korea; countryCode: KR; verbatimDepth: 88.2 m; locationRemarks: stn 35-7 #2; verbatimLatitude: 34°59'51.00"N; verbatimLongitude: 125°00'04.2"E; decimalLatitude: 34.9975; decimalLongitude: 125.00116667; **Identification:** identifiedBy: Cho, Kim & Lee; dateIdentified: 2022; **Event:** samplingProtocol: Smith-McIntyre grab; eventDate: 29/08/2020; **Record Level:** institutionID: MInRB-Hr86-L001; institutionCode: Marine Interstitial fauna Resources Bank; basisOfRecord: PreservedSpecimen
- **Type status:** Other material. **Occurrence:** individualCount: 1; sex: female; lifeStage: adult; preparations: preserved in 95% ethanol; occurrenceID: 254CB7CE-2CC4-5A76-A1B8-0018CA2FC96D; **Taxon:** kingdom: Animalia; phylum: Arthropoda; class: Copepoda; order: Harpacticoida; family: Cletodidae; genus: Stylicletodes; specificEpithet: trifidus; taxonRank: species; scientificNameAuthorship: Cho, Kim & Lee; **Location:** higherGeography: East Asia; waterBody: Yellow sea; country: Korea; countryCode: KR; verbatimDepth: 88.0 m; locationRemarks: stn 35-7 #1; verbatimLatitude: 34°59'40.14"N; verbatimLongitude: 125°00'2.82"E; decimalLatitude: 34.99448333; decimalLongitude: 125.00078333; **Identification:** identifiedBy: Cho, Kim & Lee; dateIdentified: 2022; **Event:** samplingProtocol: Smith-McIntyre grab; eventDate: 20/04/2019; **Record Level:** institutionID: MInRB-Hr86-L002; institutionCode: Marine Interstitial fauna Resources Bank; basisOfRecord: PreservedSpecimen
- **Type status:** Other material. **Occurrence:** individualCount: 1; sex: female; lifeStage: adult; preparations: dissected on 6 slides; occurrenceID: CD6754DF-B256-54AB-B7B6-B1940B774B54; **Taxon:** kingdom: Animalia; phylum: Arthropoda; class: Copepoda; order: Harpacticoida; family: Cletodidae; genus: Stylicletodes; specificEpithet: trifidus; taxonRank: species; scientificNameAuthorship: Cho, Kim & Lee; **Location:** higherGeography: East Asia; waterBody: Yellow sea; country: Korea; countryCode: KR; verbatimDepth: 91.3 m; locationRemarks: stn 35-9; verbatimLatitude: 34°59'50.04"N; verbatimLongitude: 124°30'0.36"E; decimalLatitude: 34.99723333; decimalLongitude: 124.5001; **Identification:** identifiedBy: Cho, Kim & Lee; dateIdentified: 2022; **Event:** samplingProtocol: Smith-McIntyre grab; eventDate: 20/04/2019; **Record Level:** basisOfRecord: PreservedSpecimen
- **Type status:** Other material. **Occurrence:** individualCount: 1; sex: female; lifeStage: adult; preparations: dissected on 3 slides; occurrenceID: 64AF298A-7151-5F08-8155-73F4B6494798; **Taxon:** kingdom: Animalia; phylum: Arthropoda; class: Copepoda; order: Harpacticoida; family: Cletodidae; genus: Stylicletodes; specificEpithet: trifidus; taxonRank: species; scientificNameAuthorship: Cho, Kim & Lee; **Location:** higherGeography: East Asia; waterBody: Yellow sea; country: Korea; countryCode: KR; verbatimDepth: 77.2 m; locationRemarks: stn 35-13; verbatimLatitude: 35°00'06.36"N; verbatimLongitude: 123°30'02.10"E; decimalLatitude: 35.00176667; decimalLongitude: 123.50058333; **Identification:** identifiedBy: Cho, Kim & Lee; dateIdentified: 2022; **Event:** samplingProtocol: Smith-McIntyre grab; eventDate: 17/10/2018; **Record Level:** basisOfRecord: PreservedSpecimen
- **Type status:** Other material. **Occurrence:** individualCount: 2; sex: female; lifeStage: adult; preparations: preserved in 95% ethanol; occurrenceID: 8C55C5E0-A228-559D-8DA6-3DB85121E6CC; **Taxon:** kingdom: Animalia; phylum: Arthropoda; class: Copepoda; order: Harpacticoida; family: Cletodidae; genus: Stylicletodes; specificEpithet: trifidus; taxonRank: species; scientificNameAuthorship: Cho, Kim & Lee; **Location:** higherGeography: East Asia; waterBody: Southern Sea of Korea; country: Korea; countryCode: KR; verbatimDepth: 105.8 m; locationRemarks: stn A3; verbatimLatitude: 34°25'25.01"N; verbatimLongitude: 128°29'59.34"E; decimalLatitude: 34.87361389; decimalLongitude: 128.49981667; **Identification:** identifiedBy: Cho, Kim & Lee; dateIdentified: 2022; **Event:** samplingProtocol: Smith-McIntyre grab; eventDate: 08/06/2015; **Record Level:** institutionID: MInRB-Hr86-L003; institutionCode: Marine Interstitial fauna Resources Bank; basisOfRecord: PreservedSpecimen
- **Type status:** Other material. **Occurrence:** individualCount: 1; sex: male; lifeStage: adult; preparations: preserved in 95% ethanol; occurrenceID: 543CF137-07D0-5CBC-8359-580AD73D62C2; **Taxon:** kingdom: Animalia; phylum: Arthropoda; class: Copepoda; order: Harpacticoida; family: Cletodidae; genus: Stylicletodes; specificEpithet: trifidus; taxonRank: species; scientificNameAuthorship: Cho, Kim & Lee; **Location:** higherGeography: East Asia; waterBody: Southern Sea of Korea; country: Korea; countryCode: KR; verbatimDepth: 105.8 m; locationRemarks: stn A3; verbatimLatitude: 34°25'25.01"N; verbatimLongitude: 128°29'59.34"E; decimalLatitude: 34.87361389; decimalLongitude: 128.49981667; **Identification:** identifiedBy: Cho, Kim & Lee; dateIdentified: 2022; **Event:** samplingProtocol: Smith-McIntyre grab; eventDate: 08/06/2015; **Record Level:** institutionID: MInRB-Hr86-L003; institutionCode: Marine Interstitial fauna Resources Bank; basisOfRecord: PreservedSpecimen
- **Type status:** Other material. **Occurrence:** individualCount: 3; sex: female; lifeStage: adult; preparations: dissected each on 3 slides; occurrenceID: 69D4DDFD-1995-5D4F-AB95-366C2AC433CB; **Taxon:** kingdom: Animalia; phylum: Arthropoda; class: Copepoda; order: Harpacticoida; family: Cletodidae; genus: Stylicletodes; specificEpithet: trifidus; taxonRank: species; scientificNameAuthorship: Cho, Kim & Lee; **Location:** higherGeography: East Asia; waterBody: Southern Sea of Korea; country: Korea; countryCode: KR; verbatimDepth: 56.7 m; locationRemarks: stn B3; verbatimLatitude: 34˚04'78.41"N; verbatimLongitude: 127˚30'03.13"E; decimalLatitude: 34.08844722; decimalLongitude: 127.50086944; **Identification:** identifiedBy: Cho, Kim & Lee; dateIdentified: 2022; **Event:** samplingProtocol: Smith-McIntyre grab; eventDate: 23/04/2017; **Record Level:** basisOfRecord: PreservedSpecimen
- **Type status:** Other material. **Occurrence:** individualCount: 1; sex: female; lifeStage: adult; preparations: undissected on one slides; occurrenceID: 6F688BC6-EAFE-5A16-BDEA-1E2A318A271F; **Taxon:** kingdom: Animalia; phylum: Arthropoda; class: Copepoda; order: Harpacticoida; family: Cletodidae; genus: Stylicletodes; specificEpithet: trifidus; taxonRank: species; scientificNameAuthorship: Cho, Kim & Lee; **Location:** higherGeography: East Asia; waterBody: Southern Sea of Korea; country: Korea; countryCode: KR; verbatimDepth: 80.7 m; locationRemarks: stn B4 #1; verbatimLatitude: 33˚59'53.04"N; verbatimLongitude: 127˚29'27.60"E; decimalLatitude: 33.99806667; decimalLongitude: 127.491; **Identification:** identifiedBy: Cho, Kim & Lee; dateIdentified: 2022; **Event:** samplingProtocol: Smith-McIntyre grab; eventDate: 08/06/2015; **Record Level:** basisOfRecord: PreservedSpecimen
- **Type status:** Other material. **Occurrence:** individualCount: 1; sex: male; lifeStage: adult; preparations: undissected on one slides; occurrenceID: 016028BE-DD62-5D66-B893-A6B961949873; **Taxon:** kingdom: Animalia; phylum: Arthropoda; class: Copepoda; order: Harpacticoida; family: Cletodidae; genus: Stylicletodes; specificEpithet: trifidus; taxonRank: species; scientificNameAuthorship: Cho, Kim & Lee; **Location:** higherGeography: East Asia; waterBody: Southern Sea of Korea; country: Korea; countryCode: KR; verbatimDepth: 80.7 m; locationRemarks: stn B4 #1; verbatimLatitude: 33˚59'53.04"N; verbatimLongitude: 127˚29'27.60"E; decimalLatitude: 33.99806667; decimalLongitude: 127.491; **Identification:** identifiedBy: Cho, Kim & Lee; dateIdentified: 2022; **Event:** samplingProtocol: Smith-McIntyre grab; eventDate: 08/06/2015; **Record Level:** basisOfRecord: PreservedSpecimen
- **Type status:** Other material. **Occurrence:** individualCount: 1; sex: male; lifeStage: adult; preparations: dissected on 3 slides; occurrenceID: 60111326-6851-5182-B67E-A393DFFE0C50; **Taxon:** kingdom: Animalia; phylum: Arthropoda; class: Copepoda; order: Harpacticoida; family: Cletodidae; genus: Stylicletodes; specificEpithet: trifidus; taxonRank: species; scientificNameAuthorship: Cho, Kim & Lee; **Location:** higherGeography: East Asia; waterBody: Southern Sea of Korea; country: Korea; countryCode: KR; verbatimDepth: 80.7 m; locationRemarks: stn B4 #1; verbatimLatitude: 33˚59'53.04"N; verbatimLongitude: 127˚29'27.60"E; decimalLatitude: 33.99806667; decimalLongitude: 127.491; **Identification:** identifiedBy: Cho, Kim & Lee; dateIdentified: 2022; **Event:** samplingProtocol: Smith-McIntyre grab; eventDate: 08/06/2015; **Record Level:** basisOfRecord: PreservedSpecimen
- **Type status:** Other material. **Occurrence:** individualCount: 2; sex: female; lifeStage: adult; preparations: preserved in 95% ethanol; occurrenceID: 0AF0451A-1D92-5B4B-ABA1-68448200C3F1; **Taxon:** kingdom: Animalia; phylum: Arthropoda; class: Copepoda; order: Harpacticoida; family: Cletodidae; genus: Stylicletodes; specificEpithet: trifidus; taxonRank: species; scientificNameAuthorship: Cho, Kim & Lee; **Location:** higherGeography: East Asia; waterBody: Southern Sea of Korea; country: Korea; countryCode: KR; verbatimDepth: 80.7 m; locationRemarks: stn B4 #1; verbatimLatitude: 33˚59'53.04"N; verbatimLongitude: 127˚29'27.60"E; decimalLatitude: 33.99806667; decimalLongitude: 127.491; **Identification:** identifiedBy: Cho, Kim & Lee; dateIdentified: 2022; **Event:** samplingProtocol: Smith-McIntyre grab; eventDate: 08/06/2015; **Record Level:** institutionID: MInRB-Hr86-L004; institutionCode: Marine Interstitial fauna Resources Bank; basisOfRecord: PreservedSpecimen
- **Type status:** Other material. **Occurrence:** individualCount: 4; sex: male; lifeStage: adult; preparations: preserved in 95% ethanol; occurrenceID: 757A85E8-C163-52CB-9F9A-9F35535BD59C; **Taxon:** kingdom: Animalia; phylum: Arthropoda; class: Copepoda; order: Harpacticoida; family: Cletodidae; genus: Stylicletodes; specificEpithet: trifidus; taxonRank: species; scientificNameAuthorship: Cho, Kim & Lee; **Location:** higherGeography: East Asia; waterBody: Southern Sea of Korea; country: Korea; countryCode: KR; verbatimDepth: 80.7 m; locationRemarks: stn B4 #1; verbatimLatitude: 33˚59'53.04"N; verbatimLongitude: 127˚29'27.60"E; decimalLatitude: 33.99806667; decimalLongitude: 127.491; **Identification:** identifiedBy: Cho, Kim & Lee; dateIdentified: 2022; **Event:** samplingProtocol: Smith-McIntyre grab; eventDate: 08/06/2015; **Record Level:** institutionID: MInRB-Hr86-L004; institutionCode: Marine Interstitial fauna Resources Bank; basisOfRecord: PreservedSpecimen
- **Type status:** Other material. **Occurrence:** individualCount: 1; sex: male; lifeStage: adult; preparations: dissected on 3 slides; occurrenceID: 4AA5AF24-DC37-5F71-B0DE-B10D1F7F6053; **Taxon:** kingdom: Animalia; phylum: Arthropoda; class: Copepoda; order: Harpacticoida; family: Cletodidae; genus: Stylicletodes; specificEpithet: trifidus; taxonRank: species; scientificNameAuthorship: Cho, Kim & Lee; **Location:** higherGeography: East Asia; waterBody: Southern Sea of Korea; country: Korea; countryCode: KR; verbatimDepth: 78.7 m; locationRemarks: stn B4 #3; verbatimLatitude: 33˚59'52.62"N; verbatimLongitude: 127˚29'58.20"E; decimalLatitude: 33.99795; decimalLongitude: 127.4995; **Identification:** identifiedBy: Cho, Kim & Lee; dateIdentified: 2022; **Event:** samplingProtocol: Smith-McIntyre grab; eventDate: 26/04/2016; **Record Level:** basisOfRecord: PreservedSpecimen
- **Type status:** Other material. **Occurrence:** individualCount: 2; sex: male; lifeStage: adult; preparations: preserved in 95% ethanol; occurrenceID: 6556F4AB-08CC-5649-90F4-201E34C014CF; **Taxon:** kingdom: Animalia; phylum: Arthropoda; class: Copepoda; order: Harpacticoida; family: Cletodidae; genus: Stylicletodes; specificEpithet: trifidus; taxonRank: species; scientificNameAuthorship: Cho, Kim & Lee; **Location:** higherGeography: East Asia; waterBody: Southern Sea of Korea; country: Korea; countryCode: KR; verbatimDepth: 78.7 m; locationRemarks: stn B4 #3; verbatimLatitude: 33˚59'52.62"N; verbatimLongitude: 127˚29'58.20"E; decimalLatitude: 33.99795; decimalLongitude: 127.4995; **Identification:** identifiedBy: Cho, Kim & Lee; dateIdentified: 2022; **Event:** samplingProtocol: Smith-McIntyre grab; eventDate: 26/04/2016; **Record Level:** institutionID: MInRB-Hr86-L005; institutionCode: Marine Interstitial fauna Resources Bank; basisOfRecord: PreservedSpecimen

#### Description

**Figure 2.**
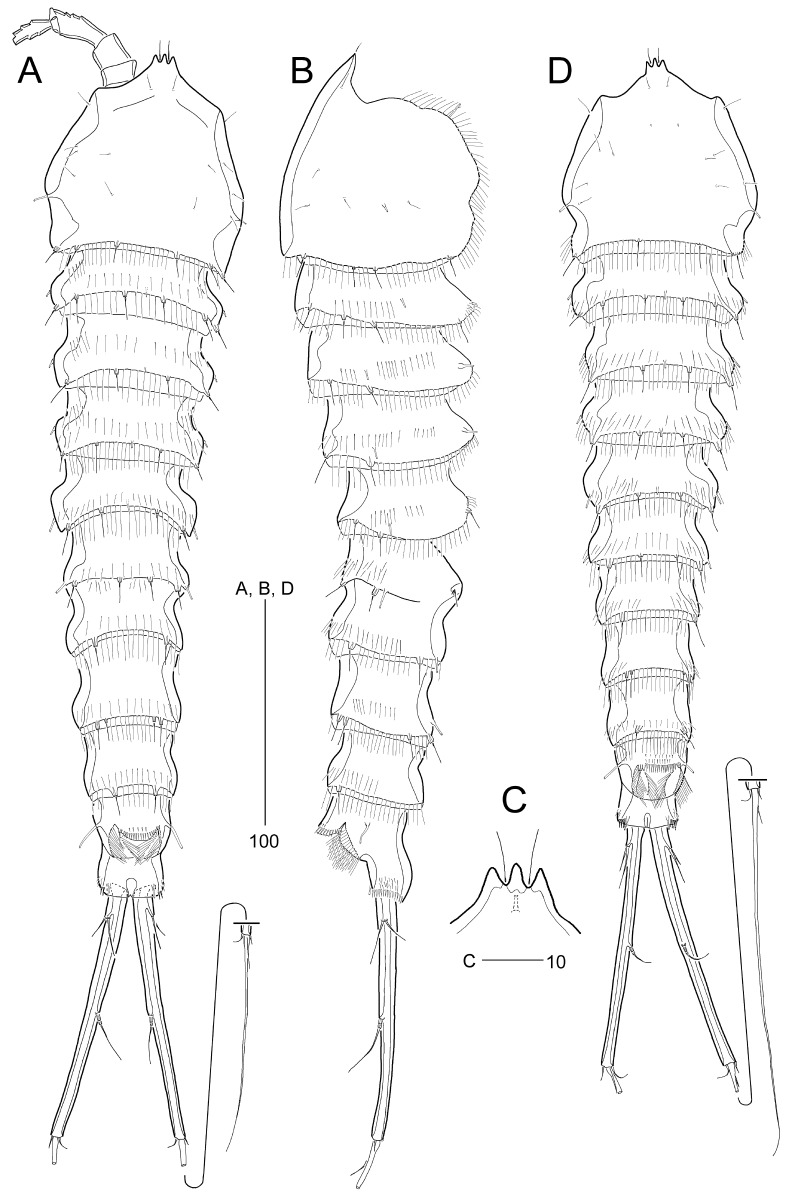
*Stylicletodestrifidus* sp. nov. Female **A** habitus, dorsal view; **B** habitus, lateral view; **C** rostrum. Male **D** habitus, dorsal view.

**Female** (based on the paratype, MABIK CR00252669): Body length 484 μm (ranging from 411–500 μm, mean = 461 μm, n= 11; holotype 457 μm). Habitus (Fig. 2A–B) cylindrical, gradually narrowing posteriorly, with unclear separation between prosome and urosome. Integument strongly chitinized, with fine, thread-like setular ornamentation, except for cephalic shield and anal somite. Prosome (Fig. 2A–B) slightly shorter than urosome, 4-segmented, comprising cephalothorax and three free pedigerous somites. Cephalothorax representing approximately 1/5 of body length, maximum width at level of posterior 2/3, with 1 pair of lateral tube pores and several pairs of sensilla; posterior margin ornamented with fine setules and with 4 pairs of sensillum-bearing socles; lateral margin with fine setules. Dorsal surface of free pedigerous somites covered with fine setules posteriorly, with 1 mid-dorsal tube pore and 1–2 pairs of lateral tube pores; posterior margins with fine setules and 2–4 sensillum-bearing socles.

**Figure 3.**
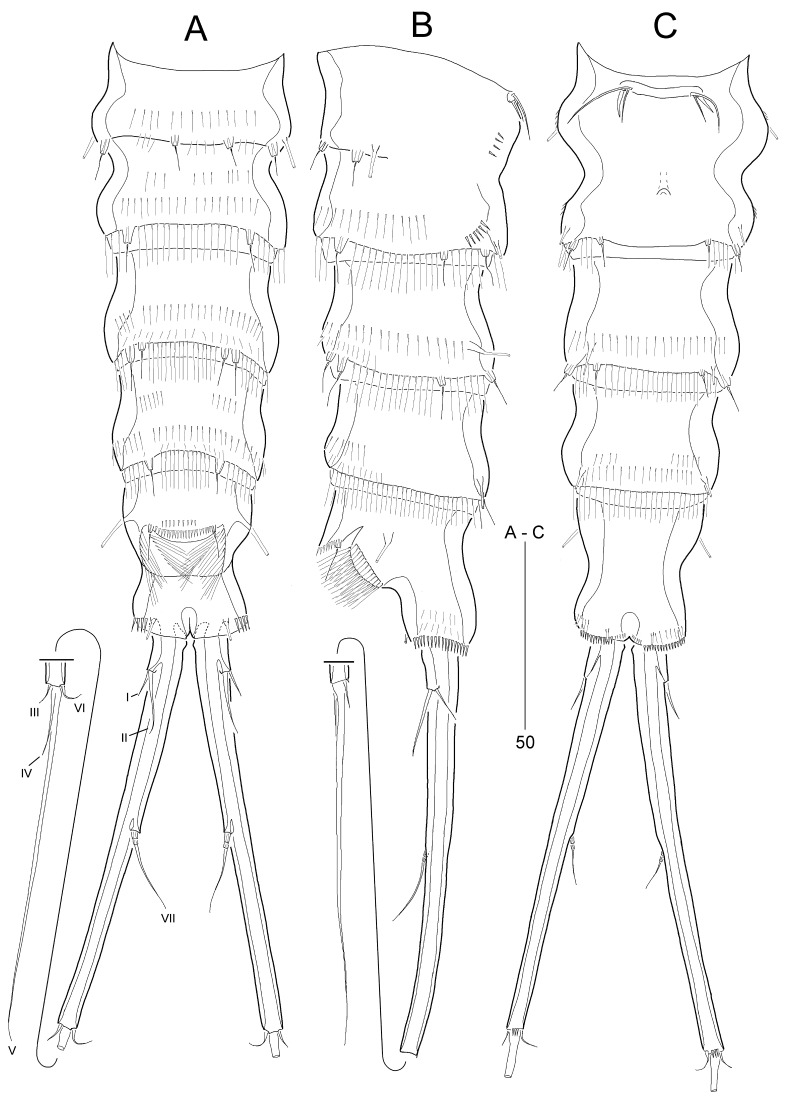
*Stylicletodestrifidus* sp. nov. Female **A** urosome, dorsal view, with setae on caudal rami numbered using Roman numerals; **B** urosome, lateral view; **C** urosome, ventral view.

Urosome (Fig. 2A–B and Fig. 3A–C) 5-segmented, comprising P5-bearing somite, genital double-somite, and 3 free abdominal somites; posterior margins, except for anal somite, as in prosome, but lacking mid-dorsal tube pore. Genital somite and third urosomite separated dorsally and laterally, but completely fused ventrally forming genital double-somite (Fig. 3A–C). Genital aperture (Fig. 3C and Fig. 8B) located at anterior half, fused medially forming a transverse slit; covered by a single plate with 3 setae representing vestigial P6 (Fig. 8B), of which innermost seta longest. Copulatory pore (Fig. 3C) located at anterior 2/3 of genital double-somite. Anal somite (Fig. 3A–C) 1.3 times longer than wide, laterally constricted at mid-length; with 3 pairs of tube pores proximally, and both subdistally in dorsal and ventral surface, respectively; ornamented with spinules laterally and ventrally along posterior border; anal opening (Fig. 3A–B) large, ornamented with long setules; anal operculum semicircular and wide, located at anterior 1/3 of anal somite, with 2 sensillum-bearing socles and 2 rows of minute dorsal spinules.

Caudal rami (Fig. 2A–B and Fig. 3A–C) distinctly divergent, cylindrical, extremely elongate, as long as three abdominal urosomites combined, L/W ratio 12.7, as long as 1/4 of body length; with few spinules at posterior margin of ramus ventrally (Fig. 3C); with 7 setae: lateral setae I and II inserted in proximal 1/8 of ramus, seta II about twice as long as seta I; seta III shortest, arising from outer distal corner; terminal seta IV fused basally to well-developed seta V, as long as seta II; terminal seta V longest, slightly shorter than ramus; seta VI short, slightly longer than seta III, located at inner distal corner; tri-articulate seta VII arising from minute dorsal pedestal, located in middle of caudal ramus.

Rostrum (Fig. 2A, C and Fig. 5B) fused to cephalic shield, reaching beyond first antennular segment; with trifid tip, with 1 pair of subapical sensilla and 1 ventral tube pore (Fig. 5B).

**Figure 4.**
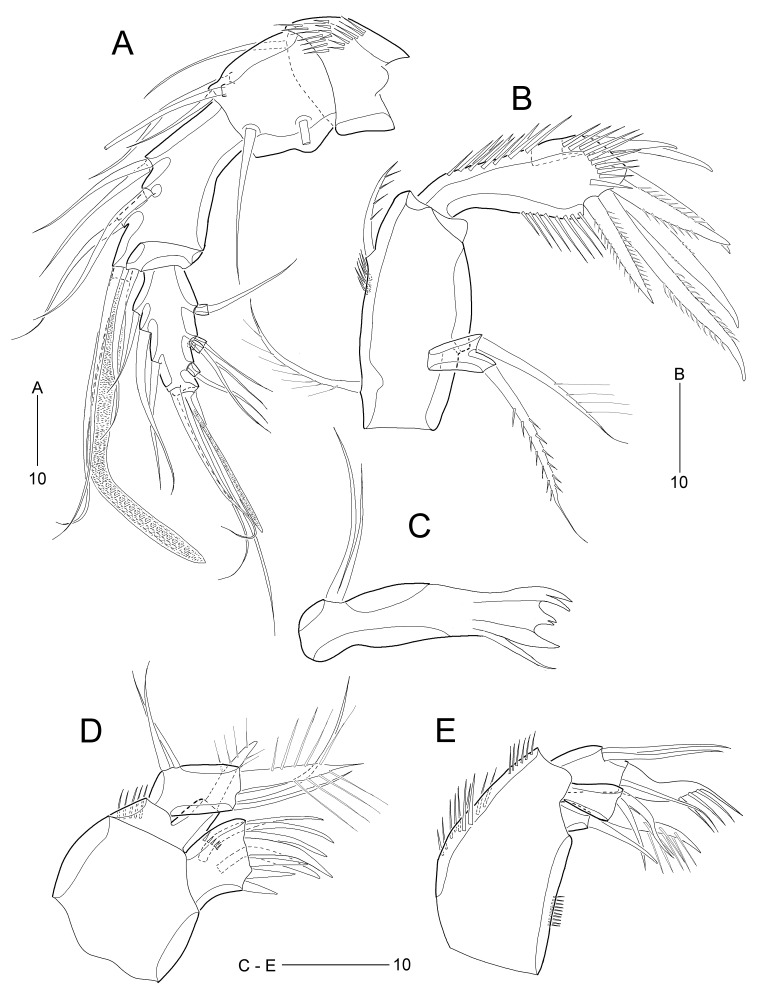
*Stylicletodestrifidus* sp. nov. Female **A** antennule; **B** antenna; **C** mandible; **D** maxillule; **E** maxilla.

Antennule (Fig. 4A) 5-segmented. First segment short, with 3 rows of inner spinules. Second segment with small, blunt process on outer margin. Third segment longest, with 1 seta fused basally to aesthetasc on distal peduncle. Fourth segment shortest. Distal segment with 5 bi-articulate outer setae; with apical acrothek composed of 1 aesthetasc and 2 long setae. Armature formula 1-[1], 2-[5], 3-[5 + (1 + ae)], 4-[1], 5-[9 + acrothek)].

Antenna (Fig. 4B). Coxa (not figured) small. Allobasis with 2 unipinnate setae and 1 group of spinules on abexopodal margin. Exopod 1-segmented, small, with 1 uniplumose and 1 pinnate seta. Free endopod ornamented with spinules along both inner and outer and subdistal margins; lateral armature composed of 2 bare spines and distal armature consisting of 1 bare, 1 weakly pinnate, and 3 pinnate spines.

Mandible (Fig. 4C). Coxa slender; gnathobase with 1 unicuspidate and 2 bicuspidate teeth and 1 bare seta (fused to coxa basally). Palp very reduced, represented by 2 naked slender setae fused at base.

Maxillule (Fig. 4D). Praecoxal arthrite strongly developed, with 2 juxtaposed bare setae on anterior surface and 6 elements around distal margin; with 1 row of posterior spinules. Coxa with 1 outer row of long spinules; endite with 1 stout plumose spine. Basis apically with 1 stout plumose spine and 2 bare setae. Endopod represented by 2 bare setae.

Maxilla (Fig. 4E). Syncoxa with 2 outer groups of strong spinules and 1 inner group of minute spinules; with 2 endites: proximal endite small, apically with 1 bare seta; distal endite longer than preceding one, with 1 stout comb-like seta and 2 bare setae. Allobasis apically with 1 stout comb-like seta and 1 slender seta. Endopod represented by 2 bare setae.

**Figure 5.**
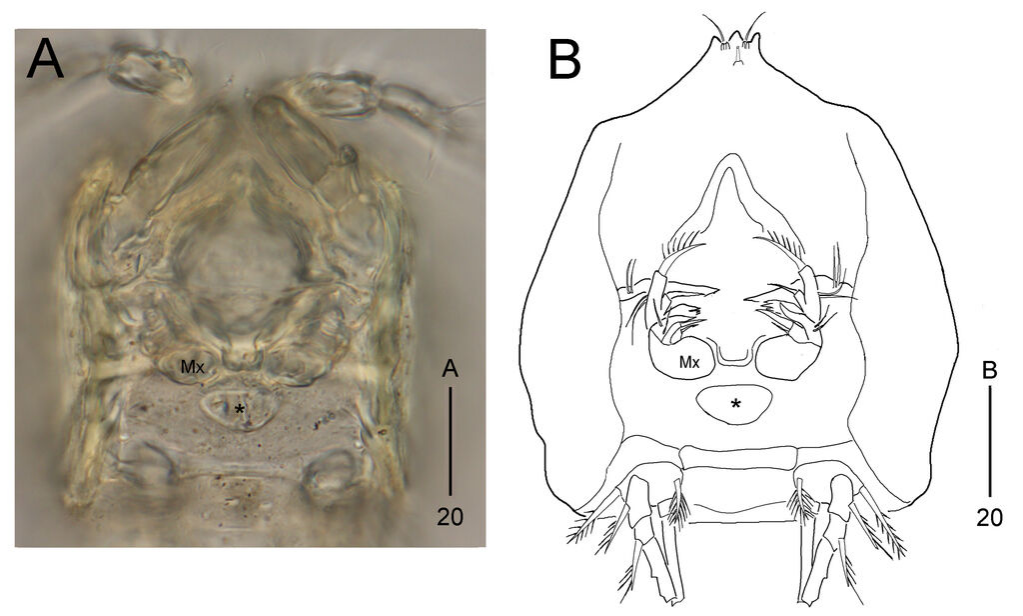
*Stylicletodestrifidus* sp. nov. Female **A** a photograph of cephalothorax by DIC microscope, ventral view; **B** a line drawing of cephalothorax, ventral view. Asterisk (*) represents the assumed position of the maxilliped and Mx indicates the position of the maxilla.

Maxilliped absent, with a small plate in the place where this appendage was supposed to be (see asterisk in Fig. 5A–B).

**Figure 6.**
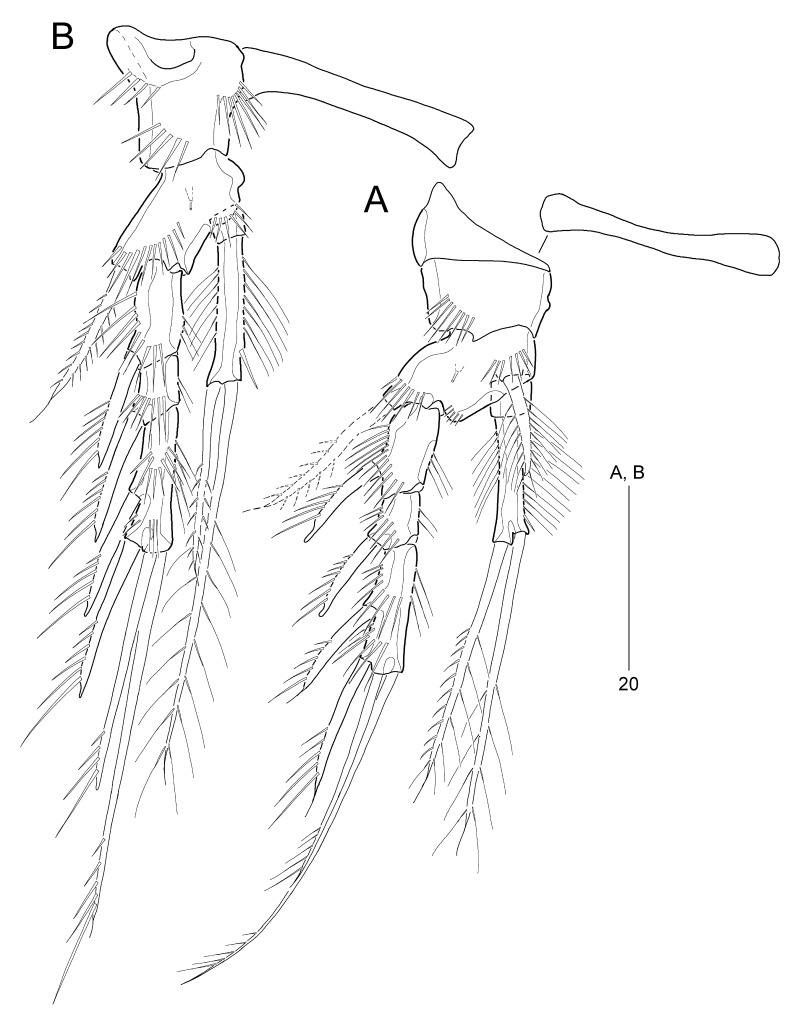
*Stylicletodestrifidus* sp. nov. Female **A** P1, anterior; **B** P2, anterior.

**Figure 7.**
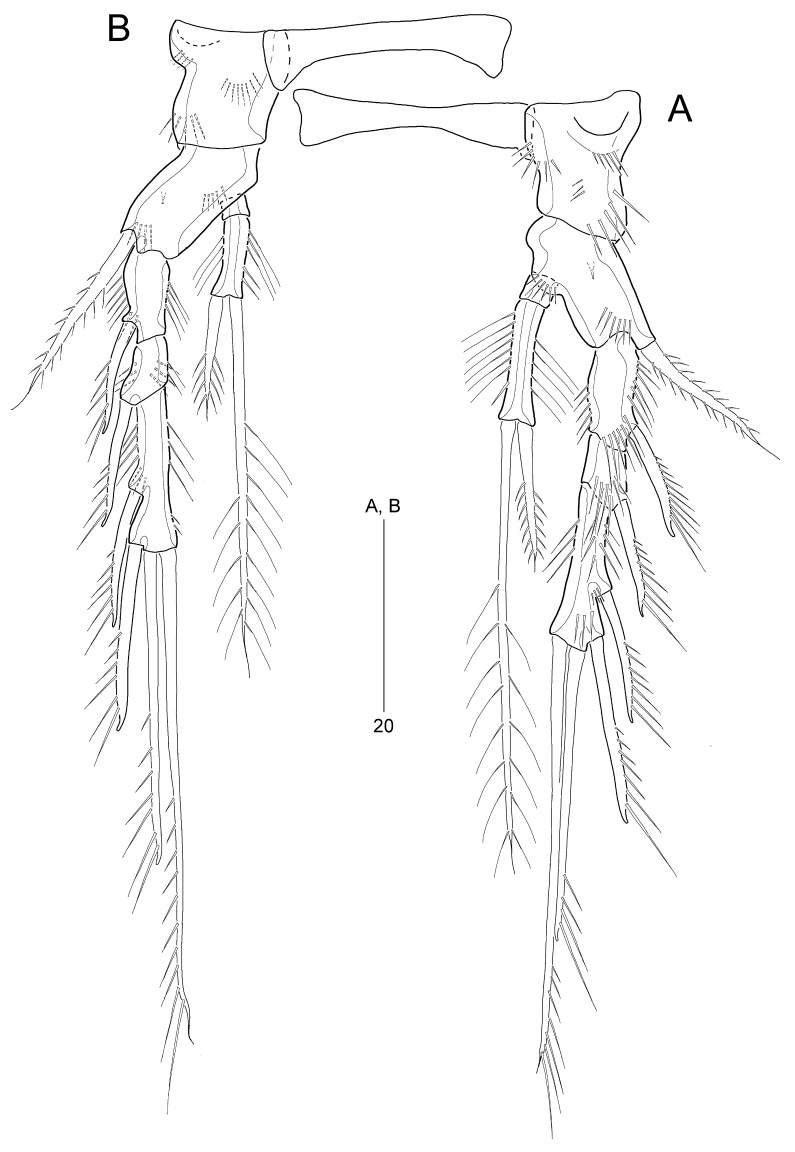
*Stylicletodestrifidus* sp. nov. Female **A** P3, anterior; **B** P4, posterior.

P1–P4 (Fig. 6A–B and Fig. 7A–B) with very wide and narrow intercoxal sclerites, without ornamentation; with 3-segmented exopods and 2-segmented endopods.

P1 (Fig. 6A). Praecoxa large, without ornamentation. Coxa with 1 anterior row of long spinules. Basis ornamented with 1 outer and 1 inner row of spinules, one additional spinular row between rami, and 1 tube pore anteriorly; with 1 long pinnate outer seta and 1 stout pinnate inner spine. Exopod longer than endopod; each segment ornamented with outer spinules and inner setules; exp-1 and exp-2 each with 1 unispinulose outer spine; exp-2 smallest; exp-3 longest with 2 unispinulose outer spines and 2 unispinulose apical setae; inner apical seta about twice as long as outer one. Endopod reaching tip of exp-2; enp-1 very small, without ornamentation and armature; enp-2 elongate, about 5 times longer than enp-1, with rows of long inner setules and thin outer spinules, 1 subdistal inner spinule, and 2 long plumose apical setae; outer apical seta ornamented with outer spinules, slightly shorter than inner one.

P2–P4 (Fig. 6B and Fig. 7A–B). Coxae and bases ornamented with anterior spinular rows; anterior surface of coxae proximally with a slight depression, strongly sclerotized. Bases weakly prolonged, with 1 pinnate outer spine and 1 anterior tube pore. Exopods longer than endopods; each segment ornamented with outer and inner spinules as figured; exp-2 shortest, exp-3 longest; exp-1 and exp-2 each with 1 unispinulose outer spine; exp-3 with 2 unispinulose outer spines and 2 unispinulose apical setae. Endopods slightly longer than exp-1 and exp-2 combined in P2, as long as exp-1 and exp-2 combined in P3, and as long as exp-1 in P4; all enp-1 without ornamentation and armature; enp-2 ornamented with outer and inner setules and a few inner spinules; P2–P3 enp-2 and P4 enp-2 about 5 and 4 times longer than enp-1, respectively; enp-2 apically with 1 pinnate (in P2) or spiniform (in P3–P4) outer seta and 1 plumose inner seta; each inner seta about 2 (P2) and 3 (P3–P4) times longer than outer seta, respectively.

Armature formula of P1–P4 shown in Table 2.

**Figure 8.**
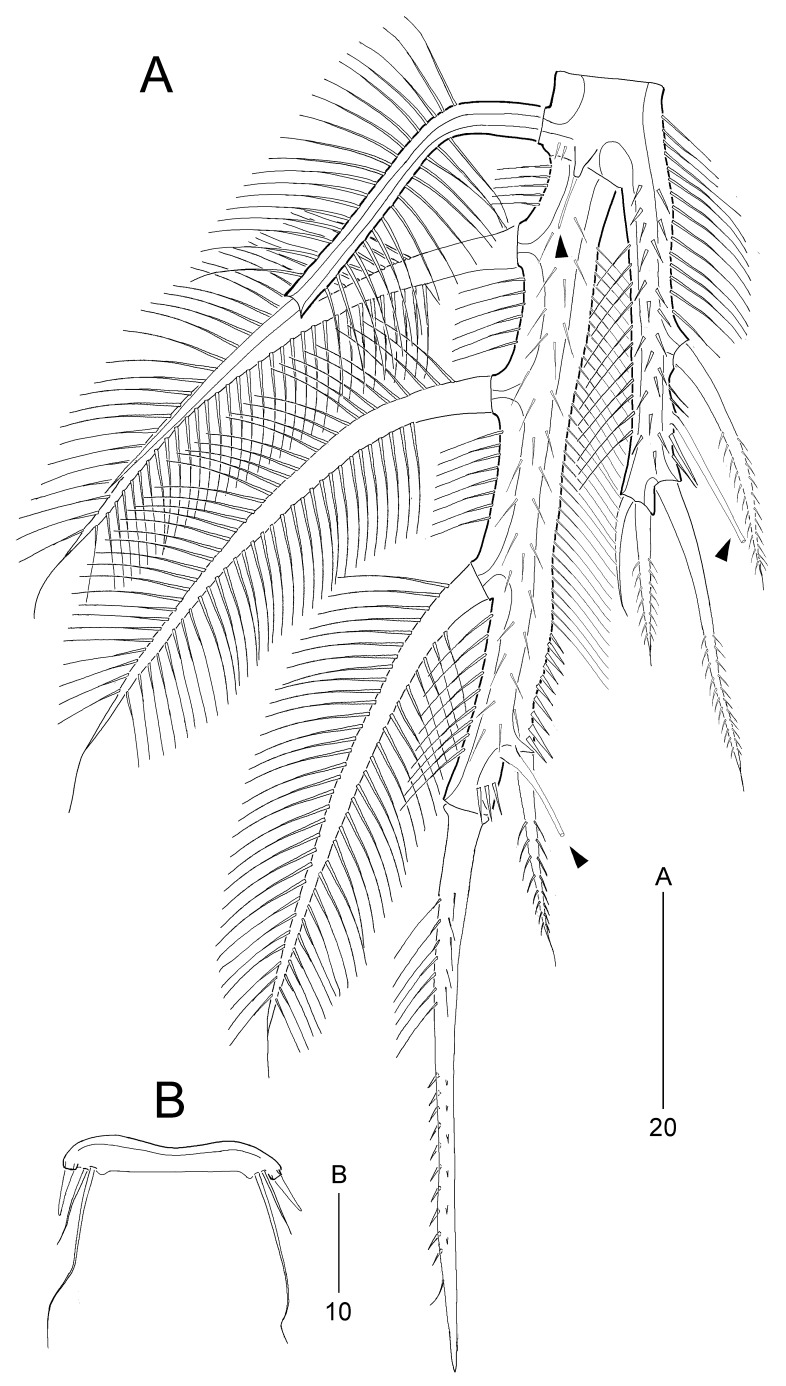
*Stylicletodestrifidus* sp. nov. Female **A** P5, anterior, arrowheads indicating tube pores; **B** P6.

P5 (Fig. 8A) consisting of baseoendopod and 1-segmented exopod. Baseoendopod with a few spinules and 1 long anterior tube pore arising from long protuberance (indicated by arrowhead in Fig. 8A); outer setophore very long, ornamented with long lateral setules, with 1 bare seta, shorter than setophore; endopodal lobe elongate, reaching middle of exopod, ornamented with anterior and lateral spinules, with 2 spiniform setae and 1 long tube pore on inner margin, and 1 bare and 1 spiniform seta on distal margin. Exopod extremely elongate, covered with anterior spinules; outer margin ornamented with spinules, with 3 densely plumose setae; inner margin ornamented with long setules proximally and minute spinules subdistally, with 1 small pinnate spine and 1 long tube pore subdistally (indicated by arrowhead in Fig. 8A); apical margin with 1 long spine ornamented with setules proximally and spinules subdistally.

**Male** (based on the paratype, MABIK CR00252670): Body (Fig. 2D) slightly smaller than female, 448 μm long (ranging from 401–475 μm, mean = 443 μm, n = 8). Sexual dimorphism in urosomal segmentation, relative length of caudal setae, antennule, P3, P5, and P6.

**Figure 9.**
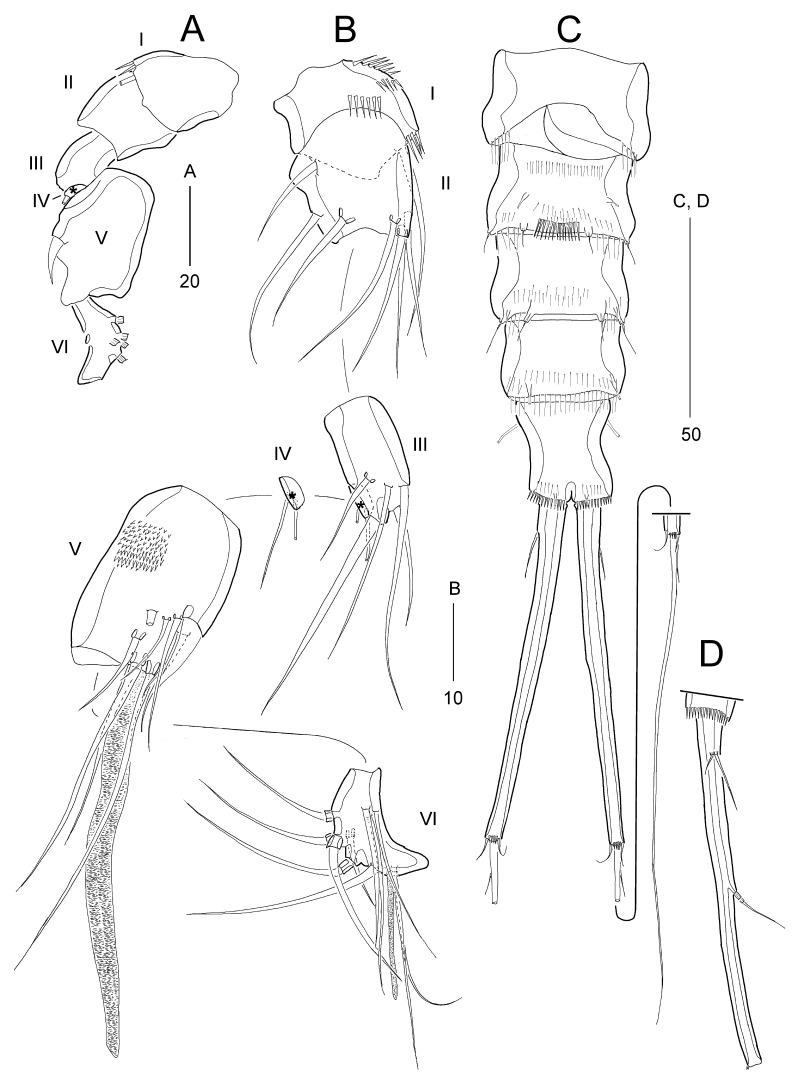
*Stylicletodestrifidus* sp. nov. Male **A** antennule, armature omitted; **B** antennule; **C** urosome, ventral view; **D** caudal ramus, lateral view, setae III–VI omitted. Asterisk (*) indicates the small fourth segment of the antennule.

Urosome (Fig. 2D and Fig. 9C) 6-segmented, genital and first abdominal somites completely separate. First abdominal somite with 1 row of ventral spinules.

Caudal rami (Fig. 9C, D) as in female, except for seta V longer than caudal ramus.

Antennule (Fig. 9A, B) 6-segmented, chirocer, with geniculation between fifth and sixth segments; with aesthetasc on fifth and sixth segments; first segment with 4 rows of inner spinules; fourth segment very small (difficult to see); fifth segment markedly swollen, with 1 anterior patch of minute spinules; sixth segment conical, slightly curved inwards. Armature formula as follows: 1-[1], 2-[7], 3-[5], 4-[2], 5-[8 + (1 + ae)], 6-[9 + acrothek].

**Figure 10.**
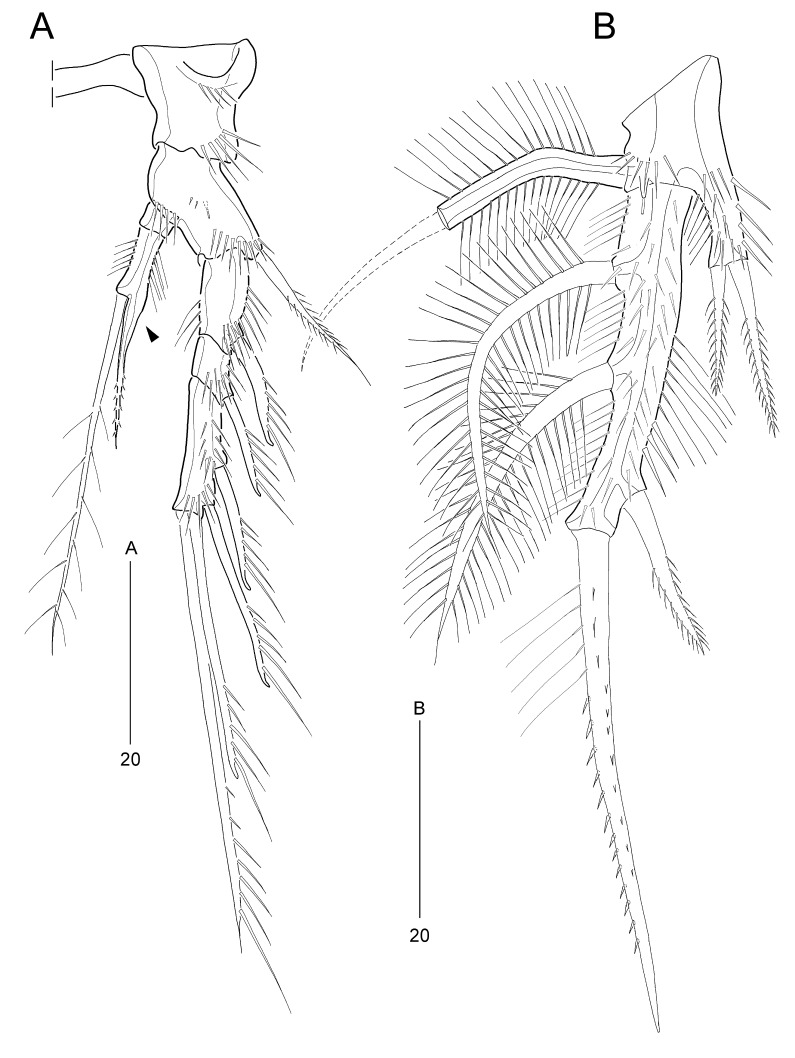
*Stylicletodestrifidus* sp. nov. Male **A** P3, anterior, arrowhead indicates the apophysis; **B** P5, anterior.

P3 (Fig. 10A). Exopod as in female; endopod 2-segmented, as in female, except for outer spine of enp-2 (indicated with an arrowhead in Fig. 10A) fused to segment forming an apophysis pinnated subdistally.

P5 (Fig. 10B) similar to female in shape, but endopodal lobe reaching proximal 1/4 of exopod, with 2 spiniform apical setae; exopod with 2 densely plumose outer setae, 1 pinnate apical spiniform element (proximally plumose) and 1 pinnate spiniform inner seta.

P6 (Fig. 9C) asymmetrical, represented by membranous flaps covering genital aperture; each lobe with 1 row of fine setules ventrolaterally.

**Variability**: Both sexes exhibited some variability in the L/W ratio of the caudal ramus (12–14.6 in females; 13.6–17.3 in males) and the L/W ratio of the exopod of P5 (7.1–10.0 in females; 5.3–7.2 in males).

**Etymology**: The specific name is derived from the Latin adjective *trifidus*, meaning “cleft into three” and refers to the characteristic shape of the rostrum with trifurcated processes at the tip. It is in the nominative singular, gender masculine.

## Discussion

The genus *Stylicletodes* was established by Lang (1936) and is a small cletodid group, currently consisting of seven valid species: *S.longicaudatus* (Brady, 1880), *S.stylicaudatus* (Willey, 1935), *S.verisimilis* Lang, 1965, *S.reductus* Wells, 1965, *S.oligochaeta* Bodin, 1968, *S.minutus* Bodin, 1968, and *S.wellsi* Ma, Liu, Li & Huys, 2021. The new species discovered in Korean waters, *S.trifidus* sp. nov., can be placed in the genus *Stylicletodes*, based on the P1–P4 exp-3 longer than exp-1 and exp-2, respectively, the outer spines of P1–P4 exopods with long outer spinules subdistally, the exopod and endopodal lobe of female P5 conspicuously elongate, and elongate caudal rami (Lang 1936, Lang 1948, Fiers 1996, Ma et al. 2021). Following the subdivision of the genus by Ma et al. (2021), *S.trifidus* sp. nov. is assigned to ‘Group I’, which includes *S.longicaudatus*, *S.oligochaeta*, *S.stylicaudatus* and *S.verisimilis*, sharing an unmodified operculum (without a median linguiform extension) and the densely plumose outer elements on the P5 exopod. However, females of *S.trifidus* sp. nov. are distinguished from the other four species by the following characters (Table 3): (1) no inner seta on P3–P4 exp-2 (absent in *S.stylicaudatus* and *S.oligochaeta*; but present in *S.longicaudatus* and *S.verisimilis*); (2) P3–P4 exp-3 with four elements (vs. five in *S.stylicaudatus* and *S.oligochaeta*; six in *S.longicaudatus* and *S.verisimilis*); (3) the P1 endopod does not reach the tip of exp-3 (vs. reaching as far as or beyond the tip of exp-3 in the other four species); and (4) the anal somite is constricted at lateral mid-length (vs. straight in the other species; the female of *S.stylicaudatus* remains unknown); longer than the wide (vs. short in *S.longicaudatus* and *S.verisimilis* and subequal in length in *S.oligochaeta*; the condition is unknown for *S.stylicaudatus*) and with the anal operculum located at anterior 1/3 of the anal somite (vs. about 2/3 in the other four species). Moreover, the antennule in the male of *S.trifidus* sp. nov. is 6-segmented consisting of an additional small segment between the third and swollen segments (Fig. 9B); it is 5-segmented in the males of other species of *Stylicletodes*. This additional segment is very difficult to observe, but recent studies of harpacticoid copepods have revealed the small segment of the male antennule under high-resolution microscopy (Lee and Huys 1999, George 2018). Therefore, it is necessary to observe the male antennule carefully.

The tip of the rostrum is either rounded or bifid in Cletodidae, with two (sub)apical sensilla and its morphology is occasionally considered a good character for identifying cletodid taxa at the genus or species levels (Gee 1994, Gee and Huys 1996, Kim et al. 2014). The rostral tip is minutely bifid in *S.longicaudatus* and *S.verisimilis* or not split in *S.stylicaudatus* and *S.wellsi*, while the rostrum of *S.oligochaeta* and *S.reductus* have not been described. *Stylicletodestrifidus* sp. nov. has a unique trifid rostrum with the sensilla between its furrows, as shown in Fig. 2C. The trifid rostrum of the new species is regarded here as apomorphic within *Stylicletodes*. This trait has been observed in a few cletodid harpacticoids, for example, *Scintisvariifurca* Por, 1986 (see Por 1986: figs. 22 and 33).

Within Cletodidae, sexual dimorphism occurs in the male P3 endopod (except for some species lacking sexual dimorphism); although Gee (1994) defined three states of the sexual dimorphism pattern in the P3 endopod, the origin of the apophysis in the males remains controversial (Gee 1994, Gee and Huys 1996, Fiers 1996). Gee (1994) postulated that the outer element on the female P3 enp-2 is homologous to the apophysis in the male, while Fiers (1996) suggested it developed from the outer hyaline membrane of the male P3 enp-2 at the last moult from the copepodid V stage to adult.

Although there are few descriptions of the males of the genus (only the males of *S.longicaudatus*, *S.stylicaudatus* and *S.wellsi* are known) (Willey 1935, Petkovski 1955, Lang 1965, Gómez 2000, Ma et al. 2021), the morphology of the male P3 endopod, including *S.trifidus* sp. nov., appears to be variable. The males of three species have a 3-segmented P3 endopod bearing a stout apophysis on the distal margin of enp-2, the third type reported by Gee (1994). In their females (except for *S.stylicaudatus*), the armature on P3 enp-2 is different, that is, *S.longicaudatus* has an outer element (spiniform) and two distal setae, whereas *S.wellsi* has only two distal setae (Table 3). Similar sexual dimorphism (it does not consider the ontogenesis) to *S.longicaudatus* was found in species of *Limnocletodes* (e.g. *L.mucronatus* Gee, 1998 in Gee (1998): fig. 4D), *Strongylacron* [e.g. *S.buchholzi* (Boeck, 1873) in Gee and Huys (1996): fig. 5C] and *Spinapecruris* [e.g. *S.curvirostris* (Scott, 1894) in Gee (2001): fig. 5B]. The form of *S.wellsi* appears in some species of *Cletodes* (e.g. *C.macrura* Fiers, 1991 in Fiers (1991): fig. 12a; *C.confusum* Gómez, 2000 in Gómez (2000): fig. 7B) and *Enhydrosoma* (e.g. *E.baruchi* Coull, 1975 in Coull (1975): fig. 4).

In comparison, the female of *S.trifidus* sp. nov. has a 2-segmented P3 endopod with an outer apical spiniform seta plus a distal seta and the male P3 endopod is 2-segmented as in the female; the outer element is enlarged and fused to the segment forming an apophysis, ornamented differently from other males of known *Stylicletodes*. The morphology of the male of *S.trifidus* sp. nov. looks similar to that of the male of *E.curticauda* (see Gee 1994: fig. 7B), *Acrenhydrosomaperplexum* (T. Scott, 1899) (as *A.perplexa* in Gee (1999): fig. 3B) and *Paracrenhydrosomanormani* Gee, 1999 (see Gee 1999: fig. 11A, G). Unfortunately, nothing is known about the development of species of *Stylicletodes* and a discussion of the sexual dimorphism of the P3 endopod will be possible only after phylogenetic studies have been conducted. Nevertheless, we suggest that the male P3 endopod morphology is a distinct apomorphy of the new Korean species in the genus.

Earlier descriptions of species of *Stylicletodes* have little or no information on the mouthparts (Brady 1880, Willey 1935, Monard 1935, Wells 1965, Lang 1965, Bodin 1968). In the description of the type species *S.longicaudatus*, Brady (1880) mentioned only the maxilliped, which (as “second pair of foot-jaws” in his text) is of ‘[moderate] size, with an ovate hand,’ but he did not provide any information about other mouth appendages. Sars (1920) first illustrated the mandible and maxilla of *S.leptostylis* (= a junior synonym of *S.longicaudatus*), but omitted the written description of the maxilliped. Later, Lang (1948) described the mouth appendages very briefly in his diagnosis of the genus *Stylicletodes* and only mentioned that the maxilliped is relatively small. Gómez (2000) and Kornev and Chertoprud (2008) (in males) provided the most detailed descriptions and illustrations of *S.longicaudatus*, although Gómez (2000) reported that the maxilliped was lost during dissection and Kornev and Chertoprud (2008) (in males) did not mention the maxilliped.

Recently, Ma et al. (2021) provided a line drawing of the maxilliped based on materials of a new species, *S.wellsi*, from the East China Sea. The maxilliped structure is very simple and unadorned and consists of the syncoxa, basis and endopod represented by a claw (see Ma et al. 2021: fig. 2F). However, both sexes of *S.trifidus* sp. nov. lack the maxilliped as shown in Fig. 5A and B, remaining as a minute single plate where this appendage is situated in other harpacticoids. In his taxonomic work, Monard (1935) also noted that he did not see the maxilliped on his specimen of *S.numidius* (= a junior synonym of *S.longicaudatus*) from Tunisia. This observation might intimate a close relationship between Korean and European specimens and some species of the genus might not retain the maxilliped. To the best of our knowledge, there have been no reports of the absence of the maxilliped in species or genera within Cletodidae and this character (without maxilliped) is unique within the family. Within harpacticoids, however, the loss of the maxilliped has rarely been documented in the genus *Leptocaris* T. Scott, 1899 of the family Darcythompsoniidae Lang, 1936 and the extreme reduction of this ramus can be observed in the genera *Cylindropsyllus* Brady, 1880 and *Cylinula* Coull, 1971 of the family Cylindropsyllidae Sars, 1909 (Huys 1988, Huys and Willems 1993). In addition, the loss of mouth parts in the male, as sexual dimorphism, appears in few deep-sea harpacticoids, such as families Aegisthidae Giesbrecht, 1893, Argestidae Por, 1986 and Pseudotachidiidae Lang, 1936 (Gómez 2018). Although the existence or non-existence of the maxilliped in all *Stylicletodes* is uncertain, this character might be important for resolving the polyphyly of *Stylicletodes* as suggested by Ma et al. (2021).

## Supplementary Material

XML Treatment for Stylicletodes trifidus

## Figures and Tables

**Table 1.** Information on sampling locations for *Stylicletodestrifidus* sp. nov. in the Yellow Sea and South Sea of Korea.

Region	Station			Date	Geographical position	Sampling depth (m)
Yellow Sea	35-7		#1	2019.04.20	34°59'40.14"N, 125°00'2.82"E	88.0
			#2	2020.08.29	34°59'51.00"N, 125°00'04.2"E	88.2
	35-9			2019.04.20	34°59'50.04"N, 124°30'0.36"E	91.3
	35-13			2018.10.17	35°00'06.36"N, 123°30'02.10"E	77.2
	37-8			2019.04.24	36°58'24.66"N, 123°48'39.72"E	77.2
South Sea	A3			2015.06.08	34°25'25.01"N, 128°29'59.34"E	105.8
	B3			2017.04.23	34°04'78.41"N, 127°30'03.13"E	56.7
	B4	#1		2015.06.08	33°59'53.04"N, 127°29'27.60"E	80.7
		#3		2016.04.26	33°59'52.62"N, 127°29'58.20"E	78.7

**Table 2.** Armature formula of swimming legs of *Stylicletodestrifidus* sp. nov.

Leg	Exopod	Endopod
P1	0.0.022	0.110
P2	0.0.022	0.020
P3	0.0.022	♀:0.020/♂:0.01apo
P4	0.0.022	0.020

**Table 3.** Main features of the females of *Stylicletodes* (the female of *S.stylicaudatus* remains undescribed and here we used the characters of the male) based on each original description. Abbreviations: A2 = antenna; AS = anal somite; CR = caudal ramus, for numbering of setae, see Fig. 3A; A2 = antenna; exp = exopod; enp = endopod; L = length; W = width; - = unknown. Note: data from figures or descriptions of each species in references.

	*S.trifidus* sp. nov.	*S.longicaudatus*	*S.verisimilis*	*S.stylicaudatus*	*S.oligochaeta*	*S.reductus*	*S.minutus*	*S.wellsi*
Sex	female	female	female	male	female	female	female	female
Body length (μm)	411–500	790	690	-	360	400	420	365–392
Rostrum	trifid	-	bifid	pointed	-	-	pointed	pointed
Position of A2 exp	proximal	-	proximal	proximal	proximal	middle	proximal	middle
No. setae on A2 allobasis	2	2	2	1	2	1	1	2
AS L:W	≒ 1.3	≒ 0.8	≒ 0.8	-	≒ 1.0	≒ 0.6	-	≒ 0.7
Operculum projection	absent	absent	absent	absent	absent	present	present	present
CR L:W	12.0–14.6	≒ 9.4	≒ 4.8	≒ 7.5	≒ 17.4	≒ 16.0	≒ 15.3	≒ 13.0
Position of seta II on CR	≒ 1/8	≒ 1/5	≒ 1/6	≒ 1/6	≒ 1/14	≒ 1/12	≒ 1/3	≒ 1/12
Position of seta VII on CR (from anterior)	≒ 1/2	-	≒ 1/3	≒ 1/4	≒ 1/2	≒ 5/6	≒ 3/5	≒ 4/5
Seta II L, reaching in CR L (from anterior)	1/4	beyond 1/2	reaching distal margin of CR	≒ 1/2	beyond 1/4	≒ 1/4	beyond 2/5	not reaching 1/2
Setae III:IV	≒ 0.3	-	≒ 1.5	≒ 2.0	≒ 0.5	-	≒ 0.5	-
Setae III:VI	≒ 0.7	≒ 1.1	≒ 2.7	≒ 2.5	≒ 0.7	≒ 1.3	≒ 0.8	≒ 1.3
CR:seta V	≒ 1.1	≒ 0.7	≒ 0.5	-	≒ 1.3	-	-	≒ 0.6
P1 exp:enp	exp>enp	exp<enp	exp<enp	exp>enp	exp>enp	exp>enp	exp≒enp	exp≒enp
	≒ 1.6	≒ 0.7	≒ 0.9	≒ 1.1	≒ 1.1	≒ 1.1	≒ 1.0	≒ 1.0
Setal formula on P1 enp	0.110	0.110	0.110	0.110	0.110^a^	0.110	0.010	0.110
Setal formula on P3 and P4 exp	0.0.022	0.1.222	0.1.222	0.0.122	0.0.122	0.0.222	0.1.122^b^	0.1.222/ 0.1.122^b^
Setal formula on P3 and P4 enp	0.020	0.021^c^	0.021	0.apo.020/ 0.021	0.021^a^	0.020^d^	0.020/ 0.010	0.020/ 0.010
P5 exp L:W	7.1–10.0	≒ 5.9	≒ 8.2	≒ 2.5	≒ 11.4	≒ 16.0	≒ 12.4	≒ 12.5
References	This study	Brady (1880)	Lang (1965)	Willey (1935)	Bodin (1968)	Wells (1965)	Bodin (1968)	Ma et al. (2021)

^a^ Bodin (1968) described the setal formula on the endopods of P1, P3 and P4 of *S.oligochaeta* as 0.020, 0.111 and 0.111, but we followed Ma et al. (2021) (437; table 2).

^b^ Ma et al. (2021) have miswritten the setal formula on the exopods of P3–P4 of *S.minutus* and P4 of *S.wellsi* as 0.1.222 in their table 2.

^c^ Brady (1880) described two apical setae on the P3 enp-2 of*S.longicaudatus* (Brady 1880: fig. 16), but other literature showed the same setal formula as that of P4 (Sars 1920, Monard 1935, Griga 1963, Lang 1965, Gómez 2000) and we followed Ma et al. (2021) (437; table 2).

^d^ Wells (1965) described the setal formula on the endopods of P3–P4 of *S.reductus* as 0.110, but we followed Ma et al. (2021) (437; table 2).
